# Antitumor Effects of Quercetin in Hepatocarcinoma In Vitro and In Vivo Models: A Systematic Review

**DOI:** 10.3390/nu11122875

**Published:** 2019-11-25

**Authors:** Paula Fernández-Palanca, Flavia Fondevila, Carolina Méndez-Blanco, María J. Tuñón, Javier González-Gallego, José L. Mauriz

**Affiliations:** 1 Institute of Biomedicine, University of León, 24071 León, Spain; pferp@unileon.es (P.F.-P.); ffonp@unileon.es (F.F.); cmenb@unileon.es (C.M.-B.); mjtung@unileon.es (M.J.T.); jgonga@unileon.es (J.G.-G.); 2 Centro de Investigación Biomédica en Red de Enfermedades Hepáticas y Digestivas (CIBERehd), 28029 Madrid, Spain

**Keywords:** combined treatments, encapsulation, flavonoid, hepatocarcinoma, quercetin, quercetin derivative

## Abstract

Quercetin is a flavonoid present in fruits, vegetables and plants with antioxidant, anti-inflammatory and anticancer properties. Its beneficial activities have been demonstrated in different human pathologies, including hepatoprotective effects against liver disorders. High mortality and late diagnosis of the primary liver tumor hepatocarcinoma (HCC) makes this cancer an interesting target for the study of quercetin effects. Our aim was to systematically review antitumor activities of quercetin in HCC preclinical studies employing single, encapsulated, combined or derived quercetin forms. Literature search was conducted in PubMed, Scopus and Web of Science (WOS), and 39 studies were finally included. We found that 17 articles evaluated quercetin effects alone, six used encapsulated strategy, 10 combined this flavonoid, two decided to co-encapsulate it and only four studied effects of quercetin derivatives, highlighting that only nine included in vivo models. Results evidence the quercetin antiproliferative and proapoptotic properties against HCC either alone and with the mentioned strategies; nevertheless, few investigations assessed specific activities on different processes related with cancer progression. Overall, further studies including animal models are needed to deeper investigate the precise mechanisms of action of quercetin as antitumor agent, as well as the potential of novel strategies aimed to improve quercetin effects in HCC.

## 1. Introduction

Quercetin (3,3′,4′,5,7-pentahydroxy flavone) is one of the main components of the polyphenol family of flavonoids [1] and it is mostly present in fruits, vegetables and some plant-derived beverages, such as wine or tea [2]. This flavonoid has many beneficial properties on human health [2], being associated its biological activity with the presence of five hydroxyl groups on the ring structure [1]. A number of studies have investigated quercetin effects on cellular processes involved in different human pathologies [3,4]. Anti-inflammatory, antioxidant and anticancer activities are some of the mainly described quercetin mechanisms of action [1,2,5]. Besides, therapeutic potential of this flavonoid has been evaluated in a broad variety of human disorders, including diabetes [3], cardiovascular [3], neurodegenerative [3,4,6] and Alzheimer’s diseases [6]; and positive actions on blood vessel pressure, intestinal microbiota and kidney disfunction [5], among others, were also related to quercetin efficacy.

Liver injury is largely caused by obesity or metabolic syndrome, in addition to high alcohol consumption [5,7]. Hepatocyte damage eventually contributes to the development of liver disorders including steatosis, alcoholic and non-alcoholic steatohepatitis which could cause non-alcoholic fatty liver disease (NAFLD), liver inflammation and hepatic fibrosis [5,7]. Hepatic chronic damage often leads to progression to liver cirrhosis and, in most cases, to hepatocarcinoma (HCC) [5,7]. In addition to the aforementioned beneficial effects, quercetin exerts multiple hepatoprotective actions through lipid biogenesis modulation, mitochondrial biogenesis activation [8] and the increase of cellular antioxidants and insulin sensitivity [5]. As part of its hepatoprotective ability, this flavonoid has demonstrated to reduce oxidative stress and inflammatory response in liver damage caused by alcohol and different toxic compounds (e.g., ethanol, metals and pesticides) [9]. Generation of an inflammatory and fibrotic microenvironment are key mechanisms produced in chronic-injured liver by hepatic stellate cells, and quercetin is able to abrogate its activation and modulate its polarization, restraining liver cells alteration [10]. Along with this, regulation of liver cell pathways involved in cell proliferation, differentiation and extracellular matrix synthesis is associated with quercetin-derived positive effects in the prevention of NAFLD [11,12] and liver fibrosis [7]. Some studies have also proved its beneficial activities against liver cirrhosis development and pulmonary associated complications [13,14], which makes quercetin a promising agent for the improvement of the outcomes in liver pathologies therapy [9].

HCC is the most common primary liver cancer and the sixth tumor with higher incidence, ranking as the fourth deadliest neoplasm worldwide [15]. Liver damage caused by different etiologic agents, mainly hepatitis C and B virus (HCV and HBV, respectively), contributes to HCC development through the stages of liver fibrosis and cirrhosis, which can take from years to decades [15]. Its complex pathogenesis and molecular heterogeneity hinder HCC early diagnosis, making curative treatments impossible [15]. In these cases, systemic therapy is used, utilizing two available tyrosine kinase inhibitors (TKIs), sorafenib and lenvatinib, in the first-line setting for advanced HCC [16]. Regardless of its effectiveness, liver cancer cells are able to develop sorafenib resistance after sustained administration [17], where several TKIs (regorafenib and cabozantinib) and monoclonal antibodies (nivolumab, pembrolizumab and ramucirumab) have been recently approved [16]. Considering toxicity and adverse reactions caused by these chemotherapeutic agents, some investigations have focused on the study of antitumor effects of natural compounds against HCC, such as resveratrol, curcumin and melatonin [18,19,20].

High mortality and treatment efficacy limitations of HCC makes it an interesting target for the study of potential antitumor effects of the natural flavonoid quercetin. Nonetheless, there are not specific review articles which comprise results from researches that evaluate properties of this flavonoid in HCC models. This systematic review is the first that summarizes quercetin antitumor activity against such liver tumor, providing clearer perspective and reliable evidences on potential use of quercetin in HCC therapy. The aim of this article was to systematically review all evidences available from in vitro and in vivo studies in which quercetin effects against HCC were analyzed, including its use as single agent as well as encapsulated, combined and derived forms of the flavonoid.

## 2. Materials and Methods

This systematic review was done based on the Preferred Reporting Items for Systematic Reviews and Meta-Analysis (PRISMA) [21].

### 2.1. Study Selection Criteria

Following criteria were used for inclusion of articles that met all of them: (i) studies that employed quercetin as single or combined agent, free or encapsulated, or quercetin-derived forms; (ii) studies that used in vitro or in vivo models of HCC; (iii) studies that reported effects directly generated by quercetin treatment.

The following criteria were used for the exclusion of articles that met any of them: (i) conference or congress communications; (ii) review articles; (iii) articles in other language than English; (iv) full articles not available; (v) studies that evaluated plant extracts effects; and (vi) studies published earlier than the last 10 years.

### 2.2. Search Strategy and Study Selection

The article search was conducted using the electronic databases PubMed, Scopus and Web of Science (WOS) on September 2019. MeSH terms used were: “quercetin AND hepatocarcinoma”, with the “[All fields]” tag in PubMed, and the fields “[Article title, Abstract, Keywords]” in Scopus and “[Topic]” in WOS search. After removing duplicates, title and abstract screening of all obtained articles was done against previously established study inclusion criteria. Selected articles were subjected to a full-text analysis excluding those that met any of the exclusion criteria. Remaining articles were considered relevant studies and were included in this systematic review.

### 2.3. Data Extraction

Data collection from each study was extracted using a standardized form and following variables were used: first author name, year of publication, quercetin administration strategy, experimental model (in vitro or in vivo), cell line or in vivo HCC induction method, general effects (e.g., antiproliferative, proapoptotic), molecular alterations (e.g., higher levels of p53, reduced G2/M population). Articles employing different samples did not allow quantitative estimates of quercetin effects in experimental HCC models. Hence, performing a meta-analysis was discarded. Data extracted from included articles were summarized and comprised in a table.

## 3. Results

### 3.1. Study Selection

**Figure 1:**
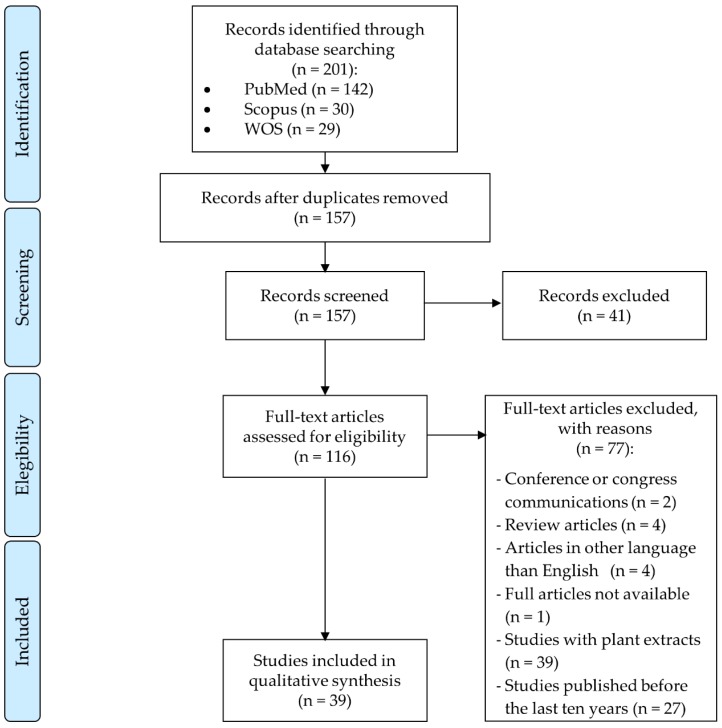
Flow diagram of the study selection process following the Preferred Reporting Items for Systematic Reviews and Meta-Analyses (PRISMA) guidelines. WOS—Web of Science.

The study selection was performed as it is described in Figure 1. A total of 201 articles were obtained in the database search, of which 29 articles were from WOS, 30 articles were from Scopus and 142 articles were from PubMed. After identifying and removing duplicates, 157 articles went under title and abstract screening and we decided to exclude 41 articles that did not meet the study inclusion criteria. Based on the study selection criteria, 116 articles were full text screened, and 77 of these articles were discarded. Finally, 39 studies met eligibility criteria and, therefore, were included in this systematic review.

### 3.2. Study Characteristics

**Figure 2:**
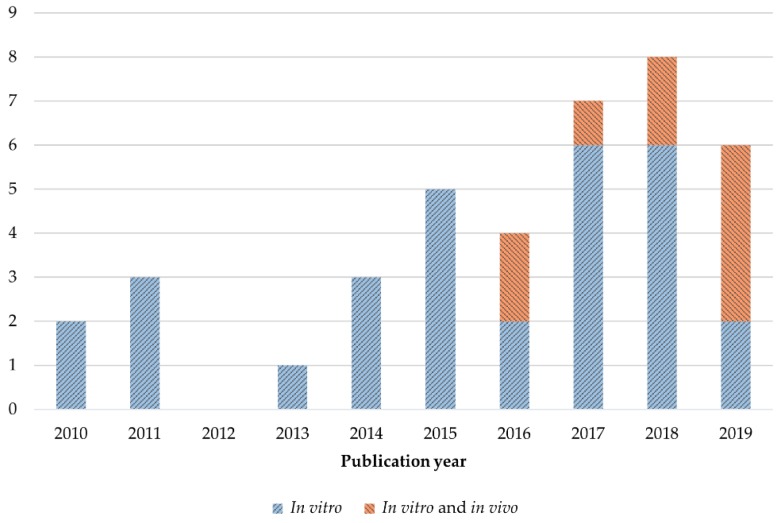
Number of articles published in the last 10 years in which quercetin effects in HCC, either as single, combined, encapsulated or derived form, were evaluated employing only cell line models in vitro or both cell and animal models in vitro and in vivo.

The main characteristics of the articles included in this review are summarized in Table 1 and Table 2. Among the 39 studies, only nine, which were published in the last four years, since 2016 (Figure 2), employed an animal model to complement the results from the in vitro assays. Six articles of the total focused on the improvement of quercetin delivery and liver tumor targeting efficiency, of which only one included in vitro and in vivo experiments. The combination of quercetin as a therapeutic strategy was studied in 10 publications, using exclusively cell lines as study model seven of them. Both strategies, quercetin encapsulation and combination, were included in two of the total articles evaluating these flavonoid effects in vitro and in vivo. Four researches of the total analyzed the effects of quercetin-derived compounds in two different HCC cell lines and the remaining 17 articles evaluated the antitumor properties of quercetin alone against HCC. The number of publications investigating this flavonoid in HCC treatment has been increasing along time (Figure 2). It has to be mentioned that out of the 39 included articles, 32 employed the HepG2 HCC line as in vitro model and only two publications used normal liver cells to observe quercetin toxicity.

### 3.3. Anticancer Activities of Quercetin in HCC Preclinical Models

#### 3.3.1. Antitumor Properties of Quercetin as Single Agent against HCC

Quercetin antitumor effects have been described in different cancer types, including HCC [1]. In 25 of the articles included in the present review, quercetin efficacy as single treatment was evaluated employing different HCC study models [22,23,24,25,26,27,28,29,30,31,32,33,34,35,36,37,38,46,48,49,51,52,53,54,57]. Antiproliferative effect of this flavonoid alone has been demonstrated in several researches with in vitro models [22,23,24,25,26,27,30,31,32,33,35,38,46,48,49,51,52,53,54], highlighting the HepG2 cell line as the most used in 21 of the 25 articles [24,25,27,28,30,31,32,33,35,36,37,38,46,48,49,50,51,52,53,54,57]. After administration of quercetin, changes in tumor cell morphology in HepG2, HuH7, PLC/PRF-5, Hep3B and LM3 lines [22,25,30,31] as well as suppression of glycolytic metabolism in SMMC-7721 and BEL-7402 HCC cell lines [23] were associated with antitumor properties in HCC. This ability to reverse glycolytic metabolism of liver cancer cells is often related to the efficacy of antitumor drugs such as quercetin [61]. Furthermore, it has been described that quercetin-derived inhibition of liver cancer cell growth could be mediated by the disruption of different pathways, including protein kinase B (Akt)/mammalian target of rapamycin (mTOR) [23,24], mitogen-activated protein kinase kinase 1 (MEK1)/extracellular signal-regulated kinase 1/2 (ERK1/2) [27,38] and Janus kinase 2 (JAK2)/signal transducer and activator of transcription 3 (STAT3) signaling routes [22]. Induction of p53 as consequence of phosphatidyl inositol 3 kinase (PI3K) and protein kinase C (PKC) downregulation was also linked to antiproliferative effects in liver tumor cells [31], in addition to the blockade of Src homology domain 2 containing tyrosine phosphatase-1/2 (SHP-1/2) activity through directly interacting with quercetin [48]. A group of researchers also observed that this flavonoid was able to abrogate the transcription factor specificity protein 1 (Sp1) expression leading to suppression of HepG2 cell proliferation [33], thus increasing the number of cellular pathways that may be altered by quercetin.

Otherwise, though cell cycle regulation is a common mechanism found to be altered in HCC cells, only eight publications have evaluated the effects of quercetin in this process, showing contradictory results [22,25,30,33,49,51,52,53]. Jeon et al. reported an increase in p53 levels and a decrease in cyclin A and checkpoint kinase 1 (CHK1) expression in HepG2 cells after quercetin treatment [25], while this drug downregulated p53 and enhanced G0/G1 and G2/M populations in HepG2 and HuH7 HCC lines [51]. Augmented levels of p21 and p27, cell cycle inhibitors, and cyclin D1 reduction were observed in another study with HepG2 cells [33] and it has been also shown that quercetin promoted G0/G1-phase arrest [49,52,53] through upregulating p16, p21 and p53 [53]. Conversely, a cell cycle arrest at G2/M phase has been described as a quercetin effect against HCC cell proliferation in HepG2 [30] and LM3 liver cancer lines [22].

Apoptosis has been clearly established as one of the mechanisms of quercetin-induced cell death in HCC [22,24,27,30,31,32,33,50,51,52,53], as it was demonstrated by the increase in proapoptotic proteins expression, such as Bax and cleaved caspases-3 and -9 [22,24,27,31,32,33,50,51,52,53], and the opposite trend in the levels of antiapoptotic proteins, for instance Bcl-2 and Mcl-1 [24,27,32,33,50,51,52]. Autophagy is a self-recycle process by which damaged cell components are degraded, and it has been associated with either pro-survival or antitumor role depending on the tumor cell context [22,62]. In this case, two studies observed that quercetin treatment promoted autophagy in LM3 [22], SMMC-7721 and HepG2 HCC cells [24], being related to apoptosis induction and suppression of tumor progression [22,24].

Angiogenesis and metastasis are tightly associated with HCC progression and represent very common targeting processes in cancer treatment [15]; however, there is only one study where quercetin effects on cell migration and invasion ability were evaluated [22]. It was reported that this flavonoid regulated the expression of epithelial and mesenchymal markers in favor of first ones, abrogating epithelial-to-mesenchymal transition (EMT) and invasiveness of LM3 HCC cells [22]. Another study has also determined a greater antiangiogenic activity of quercetin and sorafenib combination in an in vivo HCC model, but not with quercetin alone [55].

In addition to the described properties, quercetin has been shown to exert antioxidant activity in the human HepG2 cell line [25,31,57]. Nonetheless, contrary to the lower reactive oxygen species (ROS) levels observed by these three independent investigations, and the well-established antioxidant role of flavonoids [2,5], augmented ROS production was reported after quercetin administration by two different studies also in HepG2 cells [32,43]. This evidences an important need of suitable and uniform experiment design and performance to get consistent results. Among included articles, several of them determined effects of the flavonoid in different specific mechanisms [27,28,29,34,38]. These include inhibition of chymotrypsin-like activity of the proteasome, involved in proteasomal regulation of cancer signaling pathways [27,38]; rise of intracellular labile zinc, which has second messenger molecule activities in tumor cells [34]; and modulation of microRNAs expression, highly involved in cancer development and chemoresistance [63], leading to inhibition of the oncogenic RNA-binding proteins insulin-like growth factor-2 binding protein 1 and 3 (IGF2BP1 and IGF2BP3) through the upregulation of miR-1275 [29]. Another study published in 2018 employed quercetin to analyze adequacy of the cellular antioxidant (CAA) assay in HepG2 cells in order to determine the antioxidant activity of extracts from tree nuts [28].

Besides, beneficial effects of quercetin against HCC were evaluated focusing on the sensitization of chemoresistant liver cancer cells. It has been suggested that the Frizzled homolog protein 7 (FZD7)/β-catenin pathway participates in resistance mechanisms mediated by the family of ATP-binding cassette (ABC) transporters and quercetin was able to suppress it [26]. Contrariwise, the flavonoid reverted damage generated by different compounds in the HepG2 HCC cell line, including aflatoxin B_1_ (AFB_1_) [37] and ethanol-induced oxidative stress [36], or DNA lesions and genotoxicity generated after benzo[a]pyrene (B(a)P) treatment [35].

By last, despite the numerous studies conducted with quercetin, only three publications have included animal models to complete in vitro results [22,23,24]. Growth inhibitory activity of this flavonoid was demonstrated in all of them [22,23,24], describing a proapoptotic effect mediated, at least in part, by autophagy induction against LM3 and SMMC7721 HCC lines [22,24].

Altogether, the wide variety of antitumor effects of quercetin along with its demonstrated efficacy against HCC cells, set this flavonoid as a promising therapeutic agent in the treatment of HCC.

#### 3.3.2. Encapsulation for the Improvement of Quercetin Effects in HCC

Drug delivery systems have emerged as a novel mechanism of targeting cancer progression, enhancing drug efficacy through its encapsulation [42]. In this line, nanomedicine has developed numerous nanoparticles employing either organic- or inorganic-based nanocarriers [40]. Mesoporous silica nanoparticles (MSNs) conjugated with folic acid (FA) were designed to improve antitumor activity of quercetin. These quercetin-loaded nanocapsules increased cell viability inhibition of the free flavonoid and raised its antioxidant activity in an in vitro HCC model [40]. As inorganic-based carriers, gold-nanoparticles are drug delivery systems commonly chosen in nanomedicine [40]. Two independent publications synthesized poly(DL-lactide-co-glycolide) (PLGA)-loaded gold-quercetin nanoparticles to enhance quercetin efficiency in HepG2 [43], MHCC97H, Hep3B, HCCLM3 and BEL-7402 HCC cell lines [42]. Both studies found that flavonoid encapsulation decreased cell proliferation in all cell lines [42,43], being related to the blockade of several pathways, including Akt/ERK1/2, AP-2β/telomerase reverse transcriptase (hTERT) and p50/nuclear factor-κB (NF-κB)/cyclooxygenase-2 (COX-2) signaling routes in MHCC97H cells [42]. These results were also obtained in a xenograft tumor mouse model where this quercetin nano-formulation reduced in a higher extent tumor volume and weight [42]. Otherwise, cell cycle proteins expression was altered after quercetin-nanocapsules administration in both researches, finding lower levels of cyclin D1 and cyclin-dependent kinase 1 (CDK1) [42,43]. Apoptosis induction was augmented in both in vitro [42,43] and in vivo experiments [42], which was correlated with a rise in proapoptotic markers expression in HepG2 and MHCC97H cell lines [42,43], unlike the decreased levels of antiapoptotic proteins observed only in HepG2 cells [43]. In addition to cell proliferation and apoptosis, PLGA nanoparticles carrying quercetin generated morphologic alterations in both HCC lines [42,43] and inhibited MHCC97H cells migration [42]. ROS generation was higher with encapsulated flavonoid than free drug treatment and, similarly, quercetin nanoparticles diminished histone deacetylase 1 and 2 (HDAC1 and HDAC2) expression as well as HDAC activity [43]. Likewise, another group of researchers demonstrated greater cell death stimulation by encapsulating quercetin into PLGA nano-prototypes decorated with chitosan (CS) and polyethylene glycol (PEG) in HepG2 cells [41].

In vitro models of HCC have also evaluated lipid-based formulations as quercetin-encapsulation strategies [39,44]. Solid lipid nanoparticles (SLNs) containing three sterol variables (cholesterol, stigmastanol and stigmasterol) were designed and evaluated in HepG2 cell line, rising quercetin-derived cell viability reduction [44]. Similar results were reported with methoxy-poly(ethylene glycol)-b-oligo(ε-caprolactone), mPEG750-b-OCL-Bz micelles employed to co-encapsulate quercetin and superparamagnetic iron oxide nanoparticles (SPIONs) [39]. Drug resistant HepG2.2.15 cells shown lower proliferative capacity, morphological changes and G0/G1 population increment after quercetin-SPION-loaded micelles treatment respect to free quercetin administration [39].

Even though few studies analyzed quercetin nanoencapsulation as a drug delivery system in liver cancer cells, it has arisen as a novel therapeutic approach that could improve quercetin properties against HCC progression by specifically targeting tumor and increasing drug cellular uptake.

#### 3.3.3. Synergistic Effects through Quercetin Combination against HCC

Although a great number of antitumor properties of quercetin have been established in HCC treatment—mainly in cell line but also in animal models—some researchers have focused on searching for synergistic combinations with this flavonoid with the aim of improving its effectiveness against liver cancer [45,46,47,48,49,50,51,52,53,54]. Enhanced properties with the well-stablished first-line drug sorafenib was demonstrated in several HCC cell lines (HepG2, HuH7 and Hep3B2.1-7) by reducing its half-maximal inhibitory concentration (IC50) [51] and improving its tumor suppression activity [46]. Similarly, quercetin was able to raise antiproliferative action of several molecules, such as interferon-α (IFN-α) [48]; an oncolytic adenovirus expressing tumor necrosis factor-related apoptosis inducing ligand (ZD55-TRAIL) [47]; two derivatives of the organic compound maleic anhydride (3′5′-dimaleamylbenzoic acid and 3′5′-dimaleimylbenzoic acid) [49]; the chemotherapeutic drugs celecoxib [50], 5-fluorouracil (5-FU) [52] and cisplatin [53]; and the CDK inhibitor roscovitine [54]. Alterations in cell morphology of Hep3B and HepG2 HCC lines were described as results of quercetin combination with the aforementioned roscovitine [54]. Furthermore, growth inhibition ability of quercetin has been related not only to regulation of cell cycle proteins, increasing expression of p21 and p53 after cisplatin combination in HepG2 cells [53], but also with S phase arrest after individual addition of two maleic anhydride derivatives to quercetin treatment in HuH7 and HepG2 cell lines [49].

Increment of quercetin apoptosis induction was also observed after its co-administration with the following compounds, ZD55-TRAIL [47], two maleic anhydride derivatives [49], celecoxib [50], cisplatin [53] and roscovitine [54]. Moreover, increased cell death derived from quercetin and roscovitine co-treatment was dependent on Akt activation, which was disrupted by both drugs together [54].

Regulation of oxidative stress and ROS production by quercetin was also evaluated after the addition of 3′5′-dimaleamylbenzoic acid and 3′5′-dimaleimylbenzoic acid [49]. This study showed higher ROS levels after combination in comparison to quercetin alone but a reduction respect to single administration of both maleic anhydride derivatives in HuH7 HCC cell line [49]. Several signaling pathways have been related to antitumor effects of quercetin alone; nonetheless, in case of combination strategy, only JAK/STAT and NF-κB activation was found altered by quercetin addition [47,48]. Its combination with IFN-α led to greater activation of the main intermediates of JAK/STAT pathway as consequence of SHP-2 inhibition in an in vitro model of HCC [48]. Quercetin administration with ZD55-TRAIL enhanced inhibitory effects of this oncolytic adenovirus in NF-κB activation and its downstream targets p65, p50 and nuclear factor-κB inhibitor α (IκBα), which induced liver cancer cells apoptosis [47]. On the other hand, a group of researchers decided to analyze synergy between quercetin and dasatinib in cell senescence of HepG2 and HuH7 HCC cells [45]. They found that this co-treatment was not able to prevent doxorubicin-induced senescence, represented by the unaltered expression of senescent cells markers [45].

Enhancement of antiproliferative effects of quercetin in animal models was evaluated employing nude mice subcutaneously injected with HuH7 [45,47] or HepG2 cells [52]. This flavonoid increased tumor growth inhibition of 5-FU [52] and ZD55-TRAIL [47] but not that of dasatinib [45].

It should be mentioned that two independent studies put together both quercetin combination and encapsulation strategies and evaluated its cytotoxic actions both in vitro and in vivo models [55,56]. Lactoferrin shell-oily core nanocapsules coupled with lactobionic acid (LA) or glycyrrhetinic acid (GA) were designed for targeted delivery of both quercetin and sorafenib, showing greater antitumor effects in HepG2 cell line and HCC-bearing mice [55]. Similar results were described with arginine-glycineaspartic acid (RGD)-modified lipid-coated nanoparticles loaded with quercetin and sorafenib using HepG2 cells and a mouse model of HCC [56].

Globally, co-treatment strategy of quercetin with different compounds may enhance its effectiveness by mainly raising its antiproliferative and proapoptotic effects and leading to improve quercetin single-therapy properties against HCC.

#### 3.3.4. Effects of Quercetin Derivatives Treatment in HCC

Quercetin glycosides are one of the main naturally occurring forms of quercetin, which makes them interesting compounds for cancer treatment [60]. This led several researchers to analyze the effects of quercetin derivatives in different tumors, including HCC [57,58,59,60]. Cell growth inhibitory properties of quercetin-3-*O*-glucoside (Q3G) were reported in an in vitro study with HepG2 cell line, along with S and G2/M phase arrest of cell cycle and morphologic alterations [60]. This quercetin-derived compound was able to increase DNA fragmentation in parallel to apoptosis induction mediated by activation of caspase-3 and DNA relaxation activity abrogation [60]. Later, this research group decided to evaluate six long chain fatty acid esters of Q3G (stearic acid ester, oleic acid ester, linoleic acid ester, alpha-linoleic acid ester, eicosapentaenoic acid ester and docosahexanoic acid ester) in the HepG2 HCC cell line, obtaining results with the same trend that those previously published with Q3G [59].

On the other hand, 3,4-dihydroxyphenylacetic acid (DOPAC), a catabolite of some quercetin glycosides produced by colonic microflora, has been shown to augment the expression of different aldehyde dehydrogenases (ALDH1A1, ALDH2 and ALDH3A1) as well as ALDH activity in the Hepa1c1c7 mouse hepatoma cell line [58]. Besides, this catabolite induced activity on NFE2-related factor 2 (Nrf2) and aryl hydrocarbon receptor (AhR) signaling pathways aside from displaying a cytoprotective effect against acetaldehyde damage [58]. Lee et al. analyzed quercetin effectiveness in ethanol-treated HCC HepG2 cells in comparison to that of 3′-*O*-methyl quercetin (3′MQ) and quercetin-3-*O*-glucuronide (Q3GA) [57]. Results exhibited antioxidant enhanced properties of both quercetin metabolites by reverting ethanol-derived ROS accumulation, protecting from glutathione (GSH) reduction and increasing antioxidant enzymes activity [57]. These protective actions of 3′MQ and Q3GA were associated with Nrf2 activation and, in consequence, raised heme oxygenase-1 (HO-1) levels through the activator protein-1 (AP-1) transcription factor [57].

Bioactive compounds derived from quercetin have been shown to abrogate cancer progression in liver cancer cells; nonetheless, a greater number of studies would be needed to search for more quercetin derivatives and study underlying mechanisms of its antitumor action against HCC cells.

## 4. Discussion

This systematic review aimed to summarize scientific evidences concerning activities of the natural flavonoid quercetin as HCC treatment. Studies evaluating beneficial properties of quercetin encapsulation and/or combination, as well as of quercetin derivatives, were included considering its relevance in the purpose of this review. Out of the 39 included articles, 17 investigated mechanisms of signaling pathways and cellular processes alteration by quercetin alone in HCC models, including seven researches where its effectiveness as single agent besides its combination was analyzed. Although quercetin has been shown to act as a potent antitumor drug in liver cancer cell lines, only three out of the 17 publications included in vivo experiments to demonstrate its positive effects against HCC tumors. In order to accomplish greater efficiency, six studies designed quercetin-loaded nanoparticles, 10 combined this flavonoid with different compounds and two joined both strategies evaluating effects of quercetin co-encapsulation. In these cases, animal models where employed in six articles, corresponding one to flavonoid nanoencapsulation, three to its combination and the remaining two to quercetin co-encapsulation with other drugs.

Results presented in this article collect a great variety of antitumor actions of either quercetin or the mentioned strategies of combination, encapsulation and derived compounds. Cell growth inhibition, in addition to apoptosis stimulation, were the main processes described as quercetin mechanisms of action against HCC, properties that have also been observed in different tumors, such as non-small cell lung cancer [64] and breast cancer [65,66]. Although almost studies associated antiproliferative activity of quercetin with alteration of several pathways, Akt/mTOR and MEK1/ERK1/2 signaling routes were mostly found to be regulated by this flavonoid, either as free drug as well as encapsulated [23,24,27,38,42]. NF-κB-dependent pathway suppression after quercetin-loaded nanoparticles or quercetin combination therapy was also observed by two groups [42,47], however this route has not been analyzed in HCC cells treated with quercetin alone, despite the well-known role of signaling routes such as JAK/STAT and NF-κB pathways in liver cancer development [67]. Furthermore, investigations with tumor models different from HCC reported raised tumor cell growth inhibition after quercetin co-administration [68] and nanoencapsulation [69] in breast cancer, and with quercetin-derived compounds in a lymphoma cell line [70], which highlights the importance of searching for new strategies to improve quercetin effectiveness.

Blockade of cell cycle progression seems to be part of the mechanisms responsible for the efficacy of quercetin, as it was reported in HCC by different researches, and not only with quercetin [22,30,51,52,53] and its derivatives [60] but also with co-administered [49] and encapsulated forms [39]. Nevertheless, there is no consensus regarding in which phase the cell cycle arrest is induced when quercetin or the other strategies are used, described as G0/G1 [39,51,52,53], S [22,49] and G2/M population increase [22,30,51]. Contradictory results on the cell population percentage altered with quercetin were also reported in these studies, where higher G2/M cell population after quercetin administration was reported [22,30], while two different investigations found a decrease in this phase of the cell cycle [39,49]. Similarly, one study determined that quercetin-derived cell cycle detention was in S and G2/M phases in MDA-MB-231 breast cancer line [66] in contrast to the G0/G1 phase arrest observed in MCF-7 breast cancer cell line [65]. This variable role of quercetin on cell cycle regulation has also been shown in cells models of non-small cell lung cancer [64] and breast cancer [65,66], and enhanced through its combination [68], encapsulation [69], as well as with quercetin derivatives [71]. Even though quercetin was found to arrest cell cycle progression in several studies included in this systematic review, results were contradictory as opposite effects in cell population percentage were reported.

Beyond the above-mentioned tumor processes, several studies showed a great number of mechanisms modulated by quercetin. These include autophagy [22,24], oxidative stress regulation [25,31,32] and, even, protection against genotoxic agents [35,36,37]. It has to be mentioned that, contrariwise to the well-stablished antioxidant role of this flavonoid, also described in several included articles [25,31,36,37,40,49,57], two studies with in vitro models of HCC obtained that quercetin administration led to an increase in ROS formation [32,43]. Furthermore, there are few researches for each quercetin-altered process, often making it difficult to establish a specific effect, as it occurs with its antioxidant role, or in some cases prooxidant role, as in HCC. Some of these activities have been also observed in other tumors, mainly as an antioxidant effect in breast cancer cells [72]. On the other hand, it should be mentioned the low number of articles that include in vivo experiments to evaluate quercetin beneficial properties, as single agent as well as in combined, encapsulated and derived forms [22,23,24,42,45,47,52,55,56]. Among them, three investigations chose oral gavage as route of administration [22,24,45], obtaining tumor growth inhibition after quercetin treatment with doses greater than 50 mg/kg. Intraperitoneal and intravenous injection were also employed as administration routes in three and two articles, respectively, where lower quercetin doses were employed, between 10 and 50 mg/kg [23,42,52,55,56]. Only one study administered quercetin intragastrically with the higher dose of the in vivo experiments, 150 mg/kg [47]. Despite the few analyses performed with in vivo models, intermediate doses of 40 and 50 mg/kg of quercetin were those primarily employed. Moreover, only the tumor weight and volume decrease ability of quercetin were assessed, without evaluating molecular processes alterations to corroborate in vitro results. Aside from this, tumor growth inhibition of this flavonoid has been already described in other tumors employing animal models, such as in prostate cancer [73] and osteosarcoma [74].

Improvement of quercetin efficacy has become one of the purposes of some researchers, focusing on the design of nanocarriers which increase delivery efficiency and cellular uptake of this flavonoid. In the present work, six studies evaluated different nanoparticles and demonstrated higher cytotoxic effects of quercetin in both in vitro and in vivo HCC models [39,40,41,42,43,44]. This strategy has been recently arisen and numerous investigations have proved greater quercetin activities in other tumors than HCC, for example breast [75] and colon cancer [76], as well as with other compounds, such as doxorubicin and paclitaxel [77] in tumor hepatocytes. Within the included articles, some of them studied not only cytotoxicity of encapsulated quercetin but also its modulating effects on cancer-related processes and signaling pathways [42,43], providing greater information about quercetin properties. Co-treatment is a more conventional strategy to improve antitumor drug efficacy, reporting 10 publications with this HCC treatment method using quercetin [45,46,47,48,49,50,51,52,53,54]. Suppression of liver tumor cell proliferation was increased after drug combination with such flavonoid, but also some of its specific activities were potentiated, such as cell cycle arrest, antioxidant activity [49], NF-κB pathway inhibition [47] and activation of JAK/STAT signaling route [48]. As it was described with quercetin nanoencapsulation, positive effects of combination therapy have been determined in several tumors and drugs, for instance prostate cancer xenograft treated with 2-methoxyestradiol plus quercetin [78].

Natural presence of quercetin in many occurring forms in plant-derived beverages, vegetables and fruits, and its antitumor effects convert quercetin-derived molecules into interesting drugs for cancer treatment. Regardless of this, quercetin derivatives were evaluated in only four of the total articles included in this systematic review, demonstrating an HCC growth inhibitory effectiveness comparable to that of quercetin [57,58,59,60]. The potential of some of these compounds as cancer treatments were also reported in human pancreatic cancer and ovarian cancer with isoquercitrin [79] and 3,4′,7-O-trimethylquercetin [80], respectively. Despite positive results shown in different tumors by quercetin derivatives, few investigations have still focused on the study of its antitumor actions against HCC cells.

### Limitations

The limitations present in this systematic review are mainly due to the wide and heterogeneous set of articles included. Several studies reported contradictory results of oxidative stress regulation properties of quercetin as well as its specific role on cell cycle arrest, which evidences the need for homogeneity and a good design in the performance of experiments. Despite angiogenesis and metastasis are well-recognized characteristics of HCC, only two articles studied the quercetin effects in them, employing in one of them co-encapsulation strategy with sorafenib. Moreover, chemoresistance is often developed in patients with HCC; however, quercetin benefits on sensitizing tumor resistant cells was solely reported in one research. Although several publications showed a great variety of cellular processes and signaling pathways that were modulated by this flavonoid, each activity was demonstrated in one or at most two studies and in vivo model was not employed to validate the in vitro results. One of the main limitations found was the low number of in vivo experiments that were carried out, with only two researches evaluating other characteristics than tumor weight and volume decrease after quercetin treatment. Quercetin combination is a conventional strategy to improve drug efficacy; nevertheless, most articles were limited to assess only its antiproliferative properties without analyzing specific mechanisms. Lastly, few studies used quercetin derivatives to prove its positive effects in HCC.

## 5. Conclusions

In conclusion, results presented in this systematic review suggest a clear antiproliferative and proapoptotic effect of quercetin in HCC, and likely a modulating role on tumor cell cycle progression which needs to be investigated further. This flavonoid seems to have antitumoral efficacy through the alteration of a great variety of cellular processes and signaling pathways, though more studies are required to further elucidate its mechanisms of action against HCC. Arising strategies of combination and drug-delivery systems may improve such cancer inhibition properties and, along with emergent use of quercetin derivatives with anticancer efficacy, broaden the therapeutic options for HCC patients.

## Figures and Tables

**Table 1:** Basic characteristics of in vitro studies using quercetin in single, encapsulated, combined or derived forms in HCC.

First Author, Year of Publication	Quercetin Administration Strategy	Cell Line	General Effects	Molecular Alterations	Altered Signaling Pathways
Wu, 2019 [22]	Quercetin	LM3 cells	Cell viability reduction Apoptosis induction Cell cycle arrest at S and G2/M phases Autophagy induction Cell migration and invasion suppression Morphological changes	↑ Early stage apoptotic cells ↓ PCNA mRNA and protein levels ↑ Bax mRNA and protein levels ↓ Colony formation ↑ Fluorescence in TUNEL staining ↓ G0/G1 phase cells and ↑ S and G2/M phase cells ↓ Cyclin B1 protein expression ↑ E-cadherin and ↓ vimentin and MMP-9 mRNA and protein levels ↓ N-cadherin protein expression ↓ Invasiveness (Transwell invasion assay) ↓ Migrating cells (Wound-healing assay) ↑ LC3 mRNA and protein levels ↑ Beclin1 protein expression ↓ p62 mRNA and protein levels ↓ p-STAT3 protein expression ↑ LC3 protein levels decreased by IL-6 ↓ PCNA and MMP-9 protein levels enhanced by IL-6 ↓ Wound healing speed	JAK2/STAT3 inhibition
Wu, 2019 [23]	Quercetin	SMMC-7721, BEL-7402 HCC cells LO2 normal liver cells	Proliferation suppression of HCC cell lines No cytotoxic for normal hepatic cells Glycolysis inhibition	↓ Glucose uptake and lactate production ↑ 2-DG-derived cell growth inhibition ↓ HK2 mRNA and protein expression ↓ p-Akt/Akt and p-mTOR/mTOR rates	Akt/mTOR inhibition
Ji, 2019 [24]	Quercetin	SMMC-7721 and HepG2 HCC cells LO2 normal hepatic cells	Cell growth inhibition in HCC cell lines Absence of antiproliferation effect in LO2 cells Induction of autophagy Apoptosis increase	In both tumor cell lines: ↑ LC3A/LC3B-II and Beclin1 protein levels ↓ p62 protein expression In SMMC-7721: ↑ Autophagosomes and autolysosomes In all cell lines: ↓ p-Akt, p-mTOR, p-p70S6K and p-4EBP1 protein levels ↑ p-JNK, p-ERK1/2 and p-p38 MAPK protein expression ↑ Apoptotic cells percentage ↑ Bax and cleaved caspase-3 protein levels ↓ Bcl-2 protein expression	Akt/mTOR inhibition MAPK activation
Jeon, 2019 [25]	Quercetin	HepG2, HuH7, PLC/PRF-5 and Hep3B cells	Proliferation inhibition (in HepG2, PLC/PRF-5 and Hep3B cells) ROS levels reduction (in HepG2 cells) Morphological alterations	Only in HepG2 cell line: ↑ p53 and HO-1 protein expression ↓ Cyclin A and CHK1 protein levels No variation in cyclin E and SOD1 protein expression	-
Chen, 2018 [26]	Quercetin	BEL-7402 HCC cells Multidrug resistant cell line BEL/5-FU	Increase of 5-FU, MMC and ADR chemosensitivity in BEL/5-FU cells	Only in BEL/5-FU cell line: ↓ IC50 of 5-FU, MMC and ADR ↓ ABCB1, ABCC1 and ABCC2 mRNA levels ↑ Rh123 accumulation Inhibition of ABCC2 function ↑ ADR accumulation In both cell lines: ↓ FZD7, β-catenin (nuclear and cytoplasmic), ABCB1, ABCC1 and ABCC2 mRNA and protein expression	FZD7/β-catenin inhibition
Ding, 2018 [27]	Quercetin	HepG2 HCC cell line	Decrease of cell viability Apoptosis induction Inhibition of chymotrypsin-like activity	↑ TUNEL-positive cells ↑ Cleaved caspase-3, cleaved PARP and Bax protein expression ↓ Bcl-2 protein levels ↓ Chymotrypsin-like activity No changes in trypsin-like and caspase-like activities ↑ p-p38 MAPK and JNK protein expression ↓ p-ERK1/2 protein levels ↓ Protein expression of β1, β2 and β5 proteasomal subunits	MEK1/ERK1/2 inhibition
Kellet, 2018 [28]	Quercetin	HepG2 cells	Antioxidant activity	↑ CAA unit dose dependent	-
Shaalan, 2018 [29]	Quercetin	HuH7 cell line	-	↑ miR-1275 mRNA levels ↓ IGF2BP1 and IGF2BP3 mRNA expression	-
Pi, 2016 [30]	Quercetin	HepG2 cells	Suppression of cell proliferation Cell cycle arrest at G2/M phase Apoptosis increase Disruption of mitochondrial membrane potential Morphological alterations Changes in surface ultrastructure	↑ G2/M cell population ↑ Early apoptotic, late apoptotic and necrotic cells ↓ Fluorescence signal of Rh123 ↑ F-actin filaments aggregation in apoptotic cells ↑ Particle size on HepG2 membrane ↑ Surface root-mean-squared and surface average roughness ↑ Cell stiffness and Young’s modulus	-
Maurya, 2015 [31]	Quercetin	HepG2 cells	Antiproliferative activity Morphological changes	↓ ROS generation and PKC activity ↓ p-p85α, p-PKC, PKCα, COX-2 protein levels ↑ p53 protein expression and Bax mRNA levels	PI3K/p53/COX-2 and PKC/p53/COX-2 inhibition
Zhang, 2015 [32]	Quercetin	HepG2 cells	Cell viability inhibition Induction of apoptosis	Chromatin condensation and nuclei fragmentation into oligonucleosomes ↑ PIG3 mRNA and protein expression ↑ Early apoptotic cells ↑ ROS accumulation ↓ Mitochondrial membrane potential ↓ Mitochondrial cytochrome c and Bcl-2 protein expression ↑ Cytosolic cytochrome c, Bax and activated caspases -9 and -3	-
Lee, 2015 [33]	Quercetin	HepG2 cells	Decrease in cell viability Apoptosis induction	↑ Nuclear condensation and fragmentation ↑ Early and late apoptotic cells ↓ Sp1 mRNA and protein levels ↑ p21, p27, Bax, cleaved caspase-3 and cleaved PARP protein expression ↓ Cyclin D1, Mcl-1, survivin and Bcl-xL protein levels	-
Dabbagh-Bazarbachi, 2014 [34]	Quercetin	Mouse hepatoma Hepa 1-6 cell line	Augmented cytoplasmic labile zinc High ionophore activity	↑ FluoZin-3-detectable zinc ↑ Fluorescence signal of FluoZin-3	-
Kozics, 2011 [35]	Quercetin	HepG2 cells	Reduction of cell proliferation	↓ B(a)P-induced micronuclei formation and DNA damage	-
Oliva, 2011 [36]	Quercetin	Cederbaum’s CYP2E1 overexpressing HepG2 cell line	Decrease of ethanol-derived oxidative stress	↓ MDA, 4-HNE and carbonyl protein levels augmented by ethanol ↓ Ethanol-induced glutathione peroxidase 4 and SOD2 mRNA expression ↓ Gadd45b mRNA levels ↑ Nrf2 protein levels reduced by ethanol	-
Choi, 2010 [37]	Quercetin	HepG2 cells	Reduction of the AFB_1_ antiproliferative effect	↓ ROS accumulation generated by AFB_1_ ↑ AFB_1_-reduced GSH levels	-
Granado-Serrano, 2010 [38]	Quercetin	HepG2 cells	Cell proliferation suppression	↓ NF-κB and p65 nuclear translocation, NF-κB DNA-binding activity ↓ p-IκBα and IKKα protein expression ↓ Chymotrypsin-like activity No changes in trypsin-like activity ↑ DNA-binding activity of AP-1 ↑ Nuclear c-Jun levels	NF-κB inhibition AP-1/JNK activation
Srisa-nga, 2019 [39]	Quercetin encapsulation (Quercetin-SPION-loaded micelles)	HepG2.2.15 cell line	Suppression of cell growth Morphological alterations	↑ G0/G1 and ↓G2/M phase cells	-
AbouAitah, 2018 [40]	Quercetin encapsulation (FA-conjugated MSNs)	HepG2 cells	Increased antiproliferative activity	↑ Antioxidant effect ↑ Inhibition of ABTS.+ radical formation	-
Abd-Rabou, 2017 [41]	Quercetin encapsulation (CS and PEG-decorated PLGA nano-prototypes)	HepG2 cells	Cell viability reduction Apoptosis induction	↓ Quercetin IC50 ↑ Late apoptotic and necrotic cells	-
Ren, 2017 [42]	Quercetin encapsulation (PLGA-loaded gold-quercetin nanoparticles)	MHCC97H, Hep3B, HCCLM3 and BEL-7402 HCC cell lines	Decreased cell proliferation Only in MHCC97H cell line: Morphological alterations Reduction of cell migration ability Apoptosis increase	Only in MHCC97H line: ↓ Colony formation ↑ Cell-to-cell adhesions and ↓ filopodia generation and cell spreading ↓ Migrating cells ↑ P-27 protein levels ↓ c-Myc, cyclin D1, CDK1, MMP-7 and β-catenin protein expression ↑ Apoptotic cell number ↑ Cleaved caspases -9 and -3 protein levels ↑ Cytochrome c release to cytoplasm ↓ hTERT and AP-2β mRNA and protein expression ↓ hTERT promoter-binding activity of AP-2β ↓ COX-2 protein expression ↓ Binding activity of p50 on COX-2 promoter ↓ p-IKKα and p-IκBα protein levels ↑ NF-κB and p50 cytoplasm translocation from nuclei ↓ p-Akt and p-ERK1/2 protein levels	AP-2β/hTERT inhibition p50/NF-κB/COX-2 inhibition Akt/ERK1/2 inhibition
Bishayee, 2015 [43]	Quercetin encapsulation (PLGA-loaded gold-quercetin nanoparticles)	HepG2 cells	Inhibition of cell proliferation Growth rate reduction Apoptosis stimulation Morphological changes	Alteration of B-conformation of DNA ↓ p-Akt protein expression ↑ sub G-phase cells and ↓ S-phase cells ↑ p21 protein levels ↓ CDK1 and cyclin D1 protein expression ↓ HDAC activity and HDAC1/2 protein levels ↑ ROS formation ↑ rac-1 activity and later returned to basal levels Depolarization of mitochondrial membrane Bax translocation to the mitochondrial outer membrane ↑ Cytochrome c release to cytosol Generation of DNA damage ↓ Mcl-1, Bcl-2 and Bcl-xL protein levels ↑ Apaf1, caspases -9 and -3, and cleaved PARP protein expression	-
Varshosaz, 2013 [44]	Quercetin encapsulation (SLNs containing cholesterol, stigmastanol or stigmasterol)	HepG2 cells	Cell growth inhibition (the highest with cholesterol)	-	-
Kovacovicova, 2018 [45]	Quercetin combined with dasatinib	HepG2 and HuH7 cell lines	No senolytic activity exhibited	No effects in β-galactosidase activity No protein expression alteration of the senescence markers p16 and γH2A.X	-
Bahman, 2018 [46]	Quercetin	HepG2 and Hep3B cells	Antiproliferative effect	-	-
	Quercetin combined with sorafenib		Suppression of cell proliferation	-	-
Zou, 2018 [47]	Quercetin combined with ZD55-TRAIL	SMMC-7721, HepG2 and HuH7 cell lines	Decrease of cell proliferation Apoptosis induction	↑ Apoptotic bodies, nuclear fragmentation and chromatin condensation ↑ Cleaved caspases -9 and -3, cleaved PARP, Bid and Bax protein expression ↓ Bcl-2 and FLIP protein levels ↓ IκBα, p65 and p50 protein expression	NF-κB inhibition
Igbe, 2017 [48]	Quercetin	HepG2 and HuH7 HCC cell lines	Inhibition of cell viability	↓ SHP-1 and SHP-2 protein expression in HepG2 cells	-
	Quercetin combined with IFN-α		Increased cell growth inhibition in both HCC cell lines	Only in HepG2 cell line: ↓ SHP-2 protein expression ↑ p-STAT1, p-Jak1 and p-Tyk2 protein levels ↑ ISRE reporter expression ↑ 2′,5′-OAS and PKR mRNA levels ↓ Colony formation ↓ Cyclin D1 protein expression	JAK/STAT activation via SHP2 inhibition
Carrasco-Torres, 2017 [49]	Quercetin	HuH7 and HepG2 HCC cells	Antiproliferative effect Cell cycle arrest at G0/G1 phase	↑ G0/G1 cell population ↓ ROS levels and oxidized glutathione levels ↑ Reduced glutathione and GSH/GSSG index ↑ Nuclear condensation ↑ Pro-caspase-9 and cleaved caspases -9 and -3 protein expression	-
	Quercetin combined with 3′5′-dimaleamylbenzoic acid or 3′5′-dimaleimylbenzoic acid		Cell viability reduction Cell cycle arrest at S phase Antioxidant activity Apoptosis induction	In both cell lines: ↓ G2/M-phase and ↑ S-phase populations ↓ Reduced and oxidized glutathione levels and GSH/GSSG index in both cell lines (maleic anhydride derivative + quercetin) ↑ Nuclear condensation, degradation of actin and DNA ↑ Pyknotic nuclei number and TUNEL-positive cells ↑ Pro-caspase-9 and cleaved caspases -9 and -3 protein expression In HuH7 line: ↓ ROS levels In HepG2 line: ↓ ROS levels (quercetin + maleic anhydride derivative) ↑ ROS levels (maleic anhydride derivative + quercetin) ↑ Reduced glutathione levels and de novo glutathione synthesis (quercetin + maleic anhydride derivative)	-
Yu, 2017 [50]	Quercetin combined with celecoxib	HepG2 and HuH7 cell lines	Antiproliferative effect Apoptosis induction	↑ DNA fragmentation ↑ Bax protein expression ↓ Bcl-2 protein levels	-
Brito, 2016 [51]	Quercetin	HepG2, HuH7 and Hep3B2.1-7 HCC cell lines	Inhibition of cell growth and survival Apoptosis increase Cell cycle arrest	↑ Apoptotic and necrotic cells ↑ Bax/Bcl-2 ratio ↑ G0/G1 and G2/M cell population in HepG2 and HuH7 ↓ S phase cells in all cell lines ↓ p53 protein expression in HepG2 and HuH7 cells ↑ DNA damage ↑ Membrane expression of GLUT-1 ↓ Cytoplasmic expression of GLUT-1 in HepG2 and HuH7 cells ↓ ^18^F-FDG uptake	-
	Quercetin combined with sorafenib		Decrease in sorafenib IC50	-	
Dai, 2016 [52]	Quercetin	HepG2 and SMMC-7721 HCC cells	Suppression of cell proliferation Cell cycle arrest at G0/G1 phase Apoptosis increase	↑ G0/G1 phase and ↓S phase cell population ↑ Bax and Bad protein expression ↓ Bcl-2 and surviving protein levels	-
	Quercetin combined with 5-FU		Rise of 5-FU antiproliferative effects Higher apoptotic activity	-	-
Zhao, 2014 [53]	Quercetin	HepG2 cells	Inhibition of cell survival Apoptosis induction G1-phase arrest of cell cycle	↑ Cleaved caspase-3 and cleaved PARP protein levels ↑ p21, p53 and p16 protein expression ↑ G1-phase cells and ↓ S-phase cells ↑ sub-G1 cell population	-
	Quercetin combined with cisplatin		Increased growth inhibitory action Greater apoptotic effects	↑ Cleaved caspase-3 and cleaved PARP protein levels ↑ p21 and p53 protein levels	
Sharma, 2011 [54]	Quercetin	HepG2 and Hep3B cell lines	Reduced cell survival Morphological changes Apoptosis induction	↑ Apoptotic bodies ↑ p53 protein expression in HepG2 cells ↓ Pro-caspase-9 and ↑ caspase-9 protein levels in HepG2 cells	-
	Quercetin combined with roscovitine		Augmented cell proliferation inhibition Morphological alterations Apoptosis stimulation	↓ Cell density ↑ Floating cells number and apoptotic bodies ↓ p-Akt, Bcl-2 and pro-caspases -9 and -3 protein expression ↓ Bcl-2/Bax ratio and ↑ Caspases -9 and -3 protein levels	-
Abdelmoneem, 2019 [55]	Co-encapsulated quercetin and sorafenib (LF-coated, LA/LF-coated or GA/LF-coated nanocapsules)	HepG2 cells	Higher antitumoral efficacy of quercetin and sorafenib Cell viability suppression	↓ IC50 of quercetin and sorafenib ↓ Combination index ↑ Dose reduction index of quercetin and sorafenib ↑ Cellular uptake of both drugs	-
Wang, 2016 [56]	Co-encapsulated quercetin and sorafenib (RGD-modified lipid-coated nanoparticles)	HepG2 cells	Reduced cell proliferation	↓ IC50 of quercetin and sorafenib	-
Lee, 2017 [57]	Quercetin	HepG2 cells	Reduced antiproliferative action of ethanol Antioxidant activity	Reversal of ethanol effects: ↓ ROS formation ↓ MDA levels ↑ GSH, SOD and CAT levels ↑ HO-1 and nuclear Nrf2 protein expression ↑ AP-1 activity	Nrf2/HO-1 activation AP-1/HO-1 activation
	3′MQ		Lower ethanol-induced cell viability inhibition Antioxidant activity	Reversal of ethanol effects: ↓ ROS formation ↑ SOD and CAT levels ↑ HO-1 and nuclear Nrf2 protein expression ↑ AP-1 activity	Nrf2/HO-1 activation AP-1/HO-1 activation
	Q3GA		Reversion of proliferation suppression induced by ethanol Antioxidant activity	Reversal of ethanol effects: ↓ ROS formation and ↑ GSH, SOD and CAT levels ↑ HO-1 and nuclear Nrf2 protein expression ↑ AP-1 activity	Nrf2/HO-1 activation AP-1/HO-1 activation
Liu, 2017 [58]	DOPAC	Mouse hepatoma Hepa1c1c7 cell line	Reduced acetaldehyde-derived cell growth inhibition	↑ ALDH activity ↑ ALDH1A1, ALDH2 and ALDH3A1 mRNA and protein levels ↑ Nrf2 and AhR total and nuclear protein expression ↓ NF-κB nuclear expression	Nrf2 activation AhR activation NF-κB inhibition
Sudan, 2015 [59]	Six Q3G esters: Stearic acid ester Oleic acid ester Linoleic acid ester Alpha-linoleic acid ester Eicosapentaenoic acid ester Docosahexanoic acid ester	HepG2 HCC cells and normal hepatocytes	Higher cell viability of normal hepatocytes In HepG2 cells: Cell proliferation decrease Morphology changes Apoptosis induction Activity as catalytic inhibitor by DNA relaxation activity blockade	In HepG2 cells: ↓ HepG2 cell number ↑ DNA fragmentation ↑ Caspase-3 activity ↑ S and G2/M cell population ↓ G0/G1-phase cells No stabilization of topoisomerase II cleavage complexes and no formation of single linear DNA ↑ Supercoiled DNA intensity	-
Sudan, 2014 [60]	Q3G	HepG2 cell line	Cell growth suppression S-phase arrest of cell cycle Morphology alterations Apoptosis induction Catalytic inhibitor action by DNA relaxation activity inhibition	↑ S-phase and ↓ G0/G1 cell percentage ↑ DNA fragmentation ↑ Caspase-3 activity ↑ Apoptotic and necrotic cells No stabilization of topoisomerase II cleavage complexes and no formation of single linear DNA ↑ Supercoiled DNA intensity	-

^18^F-FDG: fluorine-18 fluorodeoxy-glucose; 2′5′-OAS: 2′5′ oligoadenylate synthetase; 2-DG: 2-deoxy-D-glucose; 3′MQ: 3′-*O*-methyl quercetin; 4-HNE: 4-hydroxynonenal; 4EBP1: eukaryotic translation initiation factor 4E-binding protein 1; 5-FU: 5-fluorouracil; ABCB1: ATP-binding cassette subfamily B member 1; ABCC1: ATP-binding cassette subfamily C member 1; ABCC2: ATP-binding cassette subfamily C member 2; ABTS.+: radical cations of 2,2′-azino-bis(3-ethyl-benzothiazoline-6-sulphonic acid) diammonium salt; ADR: doxorubicin; AFB_1_: aflatoxin B_1_; AhR: aryl hydrocarbon receptor; Akt: protein kinase B; ALDH: aldehyde dehydrogenase; ALDH1A1: aldehyde dehydrogenase 1 member A1; ALDH2: aldehyde dehydrogenase 2; ALDH3A1: aldehyde dehydrogenase 3 member A1; AP-1: transcription factor AP-1; Apaf1: apoptotic protease-activating factor 1; B(a)P: benzo[a]pyrene; Bad: Bcl-2-associated agonist of cell death; Bax: Bcl-2-associated X; Bcl-xL: Bcl-2-like protein 1; Bid: BH3-interacting domain death agonist; CAA: cellular antioxidant activity; CAT: catalase; CDK1: cyclin-dependent kinase 1; CHK1: checkpoint kinase 1; COX-2: cyclooxygenase-2; CS: chitosan; DOPAC: 3,4-dihydroxyphenylacetic acid; ERK1/2: extracellular signal-regulated kinase 1/2; FA: folic acid; FLIP: FLICE-like inhibitory protein; FZD7: Frizzled homolog protein 7; GA: glycyrrhetinic acid; Gadd45b: growth arrest and DNA damage-inducible protein GADD45 beta; GLUT-1: glucose transporter type 1; GSH: glutathione; GSSG: oxidized glutathione; HCC: hepatocarcinoma; HDAC: histone deacetylase; HK2: hexokinase-2; HO-1: heme oxygenase-1; hTERT: telomerase reverse transcriptase; IC50: half-maximal inhibitory concentration; IFN-α: interferon-α; IGF2BP1: insulin-like growth factor-2 binding protein 1; IGF2BP3: insulin-like growth factor-2 binding protein 3; IκBα: nuclear factor-κB inhibitor α; IKKα: inhibitor of nuclear factor-κB kinase subunit α; IL-6: interleukin 6; ISRE: interferon-sensitive response element; Jak1: Janus kinase 1; JNK: c-Jun N-terminal kinase; LA: lactobionic acid; LC3: microtubule-associated protein 1 light chain 3; LC3A: microtubule-associated protein 1A/1B light chain 3A; LC3B-II: microtubule-associated protein 1A/1B light chain 3B; LF: lactoferrin; MDA: malondialdehyde; Mcl-1: induced myeloid leukemia cell differentiation protein; MMC: mitomycin; MMP-7: matrix metalloproteinase-7; MMP-9: matrix metalloproteinase-9; MSNs: mesoporous silica nanoparticles; mTOR: mammalian target of rapamycin; NF-κB: nuclear factor-κB; Nrf2: nuclear factor erythroid 2-related factor 2; p38 MAPK: mitogen-activated protein kinase p38; p62: sequestosome-1; p70S6K: ribosomal protein S6 kinase beta-1; PARP: poly(ADP-ribose) polymerase; PCNA: proliferating cell nuclear antigen; PEG: polyethylene glycol; PIG3: p53-inducible gene 3; PKC: protein kinase C; PKR: RNA-activated protein kinase; PLGA: poly(DL-lactide-co-glycolide); Q3G: quercetin-3-*O*-glucoside; Q3GA: quercetin-3-*O*-glucuronide; RGD: arginine-glycineaspartic acid; Rh123: rhodamine 123; ROS: reactive oxygen species; SHP-1: Src homology domain 2 tyrosine phosphatase-1; SHP-2: Src homology domain 2 containing tyrosine phosphatase-2; SLNs: solid lipid nanoparticles; SOD: superoxide dismutase; SOD1: superoxide dismutase 1; SOD2: superoxide dismutase 2; Sp1: specificity protein 1; SPION: superparamagnetic iron oxide nanoparticles; STAT1: signal transducer and activator of transcription 1; STAT3: signal transducer and activator of transcription 3; TUNEL: terminal deoxynucleotidyl transferase dUTP nick end labeling; Tyk2: non-receptor tyrosine-protein kinase 2; ZD55-TRAIL: oncolytic adenovirus expressing tumor necrosis factor-related apoptosis inducing ligand.

**Table 2:** Basic characteristics of in vivo studies using quercetin in single, encapsulated, combined or derived forms in HCC.

First Author, Year of Publication	Quercetin Administration Strategy, Dose and Administration Route	Animal Model	General Effects	Molecular Alterations	Altered Signaling Pathways
Wu, 2019 [22]	Quercetin 100 mg/kg Oral gavage	Nude mice subcutaneously injected with LM3 HCC cells	Tumor growth inhibition	↓ Tumor volume (70% vs. control) ↓ Mouse weight and tumor volume ↑ Necrosis ↑ TUNEL-positive cells ↓ PCNA protein levels ↑ Bax and Beclin1 protein levels	-
Wu, 2019 [23]	Quercetin 50 mg/kg Intraperitoneal injection	SMMC-7721 xenograft mouse model	Tumor growth inhibition	↓ Tumor size ↓ HK2 and Ki67 protein expression ↓ p-Akt and p-mTOR protein levels	Akt/mTOR inhibition
Ji, 2019 [24]	Quercetin 60 mg/kg Oral gavage	Nude mice subcutaneously injected with SMMC-7221 HCC cells	Suppression of tumor growth Apoptosis and autophagy induction	↓ Tumor weight and volume ↑ LC3A/LC3B and ↓ p62 protein levels ↑ Necrosis and TUNEL staining ↑ Bax and cleaved caspase-3 protein levels ↓ Bcl-2 protein expression	-
Ren, 2017 [42]	Quercetin encapsulation (PLGA-loaded gold-quercetin nanoparticles) 30, 40 and 50 mg/kg Intraperitoneal injection	MHCC97H xenograft mouse model	Suppression of tumor growth and progression Apoptosis increase	↓ Tumor weight and volume ↓ AP-2β and COX-2 protein levels ↑ TUNEL-positive cells ↓ Cleaved caspases -9 and -3, cytoplasmic cytochrome c, p-IKKα, p-IκBα, p-NF-κB, p50, hTERT, p-Akt, Raf, and p-ERK1/2 protein expression	AP-2β/hTERT inhibition p50/NF-κB/COX-2 inhibition Akt/ERK1/2 inhibition
Kovacovicova, 2018 [45]	Quercetin combined with dasatinib 50 mg/kg of quercetin with 5 mg/kg of dasatinib Oral gavage	Mice subcutaneously injected with HuH7 cells	Absence of tumor growth inhibition	-	-
Zou, 2018 [47]	Quercetin combined with ZD55-TRAIL 150 mg/kg of quercetin with 1 × 10^9^ plaque-forming units of ZD55-TRAIL Intragastrical injection of quercetin and intratumor injection of ZD55-TRAIL	HuH7 xenograft mouse model	Tumor growth inhibition	↓ Tumor volume	-
Dai, 2016 [52]	Quercetin 40 mg/kg of quercetin with 30 mg/kg of 5-FU Intraperitoneal injection	Nude mice subcutaneously injected with HepG2 HCC cells	Decreased tumor progression	↓ Tumor volume	-
	Quercetin combined with 5-FU 40 mg/kg of quercetin with 30 mg/kg of 5-FU Intraperitoneal injection		Higher tumor growth inhibition	↓ Tumor volume	-
Abdelmoneem, 2019 [55]	Co-encapsulated quercetin and sorafenib (LF-coated, LA/LF-coated or GA/LF-coated nanocapsules) 10 mg/kg of quercetin and sorafenib Intravenous injection	DEN-induced HCC in a rat model	Antiangiogenic activity Apoptosis induction Liver weight reduction	↓ NF-κB and TNF-α mRNA expression ↓ VEGF and Ki67 protein expression ↑ Caspase-3 protein expression ↓ ALT levels by LF-coated nanocapsules ↓ ALT, AST and RLW levels by LA/LF-coated and GA/LF-coated nanoparticles Improved histological features	NF-κB inhibition
Wang, 2016 [56]	Co-encapsulated quercetin and sorafenib (RGD-modified lipid-coated nanoparticles) 40 mg/kg of quercetin alone In combination: 20 mg/kg of quercetin with 10 mg/kg of sorafenib Intravenous injection	HepG2 xenograft mouse model	Tumor progression suppression	↓ Tumor volume	-

5-FU: 5-fluorouracil; Akt: protein kinase B; ALT: alanine aminotransferase; AST: aspartate aminotransferase; Bax: Bcl-2-associated X; COX-2: cyclooxygenase-2; DEN: diethylnitrosamine; ERK1/2: extracellular signal-regulated kinase 1/2; GA: glycyrrhetinic acid; HCC: hepatocarcinoma; HK2: hexokinase-2; hTERT: telomerase reverse transcriptase; IκBα: nuclear factor-κB inhibitor α; IKKα: inhibitor of nuclear factor-κB kinase subunit α; LA: lactobionic acid; LC3A: microtubule-associated protein 1A/1B light chain 3A; LC3B: microtubule-associated protein 1A/1B light chain 3B; LF: lactoferrin; mTOR: mammalian target of rapamycin; NF-κB: nuclear factor-κB; p62: sequestosome-1; PCNA: proliferating cell nuclear antigen; PLGA: poly(DL-lactide-co-glycolide); RGD: arginine-glycineaspartic acid; RLW: relative liver weight; TNF-α: tumor necrosis factor-α; TUNEL: terminal deoxynucleotidyl transferase dUTP nick end labeling; VEGF: vascular endothelial growth factor; ZD55-TRAIL: oncolytic adenovirus expressing tumor necrosis factor-related apoptosis inducing ligand.
